# Sparse graphs-based dynamic attention networks

**DOI:** 10.1016/j.heliyon.2024.e35938

**Published:** 2024-08-08

**Authors:** Runze Chen, Kaibiao Lin, Binsheng Hong, Shandan Zhang, Fan Yang

**Affiliations:** a Department of Computer Science and Technology, Xiamen University of Technology, Xiamen, 361024, China; b Department of Automation, Xiamen University, Xiamen, 361005, China

**Keywords:** Graph neural networks, Graph attention networks, Sparse graphs, Dynamic attention

## Abstract

In previous research, the prevailing assumption was that Graph Neural Networks (GNNs) precisely depicted the interconnections among nodes within the graph's architecture. Nonetheless, real-world graph datasets are often rife with noise, elements that can disseminate through the network and ultimately affect the outcome of the downstream tasks. Facing the complex fabric of real-world graphs and the myriad potential disturbances, we introduce the Sparse Graph Dynamic Attention Networks (SDGAT) in this research. SDGAT employs the $L_{0}$ regularization technique to achieve a sparse representation of the graph structure, which eliminates noise and generates a more concise sparse graph. Building upon this foundation, the model integrates a dynamic attention mechanism, allowing it to selectively focus on key nodes and edges, filter out irrelevant data, and simultaneously facilitate effective feature aggregation with important neighbors. To evaluate the performance of SDGAT, we conducted experiments on three citation datasets and compared its performance against commonly employed models. The outcomes indicate that SDGAT excels in node classification tasks, notably on the Cora dataset, with an accuracy rate of 85.29%, marking a roughly 3% enhancement over the majority of baseline models. The experimental findings provide evidence that SDGAT delivers effective performance on all three citation datasets, underscoring the efficacy of the dynamic attention network built upon a sparse graph.

## Highlights

• Sparse graph-based dynamic attention module reduces model's reliance on original structure.
• Integration of dynamic attention and sparse graph structure optimizes data aggregation in each layer's attention mechanism.
• SDGAT performed well in node classification experiments.

## 1 Introduction

Graphs are frequently utilized to represent real-world datasets across a multitude of studies. For instance, within social networks, a graph effectively illustrates the social ties among individuals, with each node symbolizing a person and the edges denoting connections like friendships, shared interests, or interactions. Efficient representations of graph data are essential, exerting a direct impact on the outcomes of downstream tasks [1], [2]. As a powerful tool for deep representation learning to analyze graph data, Graph Neural Networks (GNNs) has gained significant attention recently and has diverse applications in fields such as node classification [3], graph classification [4], link prediction [5], and graph matching [6].

The practical application of GNNs faces a multitude of challenges. Previous studies on GNNs frequently presupposed the utilization of accurate graph structures. Nonetheless, in real-world scenarios, these graph structures are often derived from intricate interaction systems that are inherently fraught with uncertain information or errors. As a result, this assumption is often found to be inaccurate [7]. Moreover, real-world graph data often contains noise, which has a significant impact on the performance of GNNs in processing graph-structured data [8], [9], [10]. To summarize, given the widespread presence of noise in graph data, it is essential to strive to reduce the dependence of GNNs on graph structures, as this raises questions about the accuracy of GNNs in processing such data and the reliability of their results [11].

Graph sparsification is a crucial technique in obtaining a more coherent graph structure by eliminating excess graph structure noise. Traditional approaches to sparsification generally involve pruning the weight matrix and can be categorized as either structural pruning [12] or unstructured pruning [13]. Structural pruning entails the removal of specific rows or columns from a weight matrix to achieve a desired structure, often resulting in a sparse matrix. This approach results in a model that is both more succinct and computationally efficient. Unstructured weight pruning directly eliminates specific elements from the weight matrix, resulting in a high level of sparsity in the remaining matrix. Nonetheless, these conventional sparsification methods come with several inherent limitations. First, the pruned sparse model frequently shows reduced performance relative to the original, requiring some retraining to regain its initial efficacy [14]. Second, conventional sparsification methods often conduct weight-based pruning without considering the significance of each weight. This approach may inadvertently eliminate critical weights, thereby affecting the model's overall performance [15]. The aforementioned analysis illustrates the limitations of traditional sparsification methods, particularly in terms of model performance. In recent years, sparsity techniques have been extensively employed in the domain of graph neural networks. However, existing sparsity techniques have certain shortcomings. For instance, The NeuralSparse model, introduced by Zheng et al. [16], relies on a single sampling technique to sparsely process the graph. In contrast, Zhang et al. [17] have developed the Adaptive Node Sampling for Graph Transformer (ANS-GT), which can adaptively select from a variety of sampling methods to further mine the information within the graph. However, both methods lack the capability to eliminate noise from the graph. Hence, the novel randomized deletion process utilized by Fang et al. [18] in the DropMessage directly deletes messages in transit for removal. Nonetheless, this method may lead to the removal of essential information, thus affecting the model's performance. To address these issues, this research aims to alleviate graph structure noise by utilizing binary masks to retain essential information and overcome the limitations of traditional sparsification methods.

GNNs update the state of a node by interacting with its neighboring nodes, and different GNN variants employ various approaches to aggregate the representations of neighboring nodes with their own representations [19], [20], [21]. The Graph Attention Network (GAT), a widely utilized GNN architecture [22], employs an attention mechanism for aggregating neighbors, thus facilitating adaptive weighting of different neighboring nodes. Additionally, GAT employs multiple attention heads to compute multiple sets of attention coefficients for aggregation, significantly enhancing the expressive capability of graph neural network models. Consequently, GAT has gained extensive acknowledgment as one of the cutting-edge neural architectures in the domain of graph learning [23]. Traditional GAT models employ a static attention mechanism, where the attention weights between a given node and its neighbors are constant [24], [25]. For each node, the attention weights of its neighboring nodes are computed using a predefined attention function that depends on the scores of those neighbors. In other words, the nodes themselves do not influence the scoring of the attention coefficients, resulting in equal ranking of any node receiving an attention coefficient. However, this approach restricts the expressive capacity of GAT since it lacks the ability to dynamically adjust the attention mechanism based on the specific requirements of nodes, thereby hindering the capture of the intricate dynamics in the relationships between nodes.

**Motivation.** Noise exists within real graph structures, exerting a substantial impact on downstream tasks. While graph sparsity is an important method for removing noise from graph structure, its traditional model still has great room for improvement in terms of performance, and there are certain shortcomings in recent sparsity-related research that cannot remove noise in a more targeted and efficient way. Although the Sparse Graph Attention Networks (SGAT) proposed by Ye and Ji [26] utilize $L_{0}$ norm regularization to sparsify the graph, thereby optimizing the aforementioned issues, it relies on traditional static attention mechanisms. This static attention mechanism severely constrains its expressive capacity, and existing studies on graph sparsity have not effectively addressed the limitations of the static attention mechanism.

To tackle the aforementioned challenges, diminish the model's reliance on graph structure, and bolster the extraction of pertinent node interactions, we propose the Sparse Graph Dynamic Attention Networks (SDGAT). By incorporating the dynamic attention mechanism model upon sparsification of the graph structure, the model gains access to richer insights into the interactions among diverse nodes within a rational and succinct graph framework. SDGAT achieves graph sparsity through $L_{0}$ norm regularization, aiming to minimize edge noise and enable the graph neural network to be optimized, thereby emphasizing the relevant edges within the graph. To enhance the model's robustness further, a dynamic attention module is devised accordingly. Dynamic attention possesses the capability to flexibly concentrate on pivotal nodes and edge information, thereby affording increased adaptability. Moreover, dynamic attention aids the model's attention mechanism in capturing the interactions between diverse nodes more effectively, thereby enhancing the model's generalization capacity.

**Contributions.** SDGAT is a graph learning algorithm that reduces computational cost, lessens dependence on graph structure, and maintains robustness. This research has several contributions:

(1) To tackle the intricacy of real graph structures, a dynamic attention mechanism module is designed, based on the sparse graph structure. It effectively filters out noise within the graph structure while proficiently capturing the interplay information among distinct nodes.
(2) The conducted ablation experiments have conclusively shown that the combination of dynamic attention and sparse graph structure can lead to more effective information for each layer of attention.
(3) Node classification experiments on actual datasets demonstrate that SDGAT enhances performance by 2-5% on the Cora dataset in comparison to other baselines and outperforms SGAT in all datasets. To further validate the effectiveness of SDGAT, this research also executed ablation experiments, parameter sensitivity analysis and created visualization.

## 2 Related work

This chapter commences with an introduction to the foundational concepts of graph neural networks, provides a succinct overview of associated technologies, and then discusses the current graph sparsity technology in detail from four aspects. To conclude, the theoretical basis of the graph attention mechanism involved in this paper is expounded.

### 2.1 Graph neural network

In recent years, the investigation into GNN has been incessant, establishing it as one of the most rapidly burgeoning research domains in the fields of machine learning and deep learning [27], [28], [29]. GNN exhibits a wide array of applications, encompassing areas such as natural language processing [30] and computer vision [31], [32]. As an indispensable constituent of GNNs, spectral methods procure node representations that are contingent upon graph theory. One of the initial spectral-based convolutional networks for graphs employed the Fourier domain [33]. For graphs with low dimensions, GNN employs convolutional layers with multiple parameters that are invariant to the input size, thereby generating an efficient deep architecture. ChebNet [34] employs Chebyshev polynomials as approximations to the Laplace matrix, negating the need for costly eigenvalue decomposition operations. Based on this foundation, the proposed Graph Convolutional Networks (GCN) further streamlines the aforementioned approach by employing a first-order approximation of Chebyshev polynomials [35]. GAT enhances GCN by using the attention mechanism to perform aggregation operations on neighboring nodes [24]. Several other iterations have been developed based on GCN and GAT, such as Multi-feature Fused Collaborative Attention Network [36], which dynamically integrates temporal and positional features to measure their individual contributions. Subsequently, it incorporates the amalgamation of multiple features into inputs for a unified collaborative attention network, facilitating the capture of collaborative signals at higher orders. Neighbor Graph Aggregated Graph Attention Network [37] leverages multi-level sampling techniques to delineate the intricate neighborhood structures and their corresponding graphs, capturing the nuanced node-to-node interactions, while incorporating hierarchical attention mechanisms to dynamically assess the significance of different entities.

### 2.2 Graph sparsification

Graph sparsification is a prominent research domain within the realm of graph neural networks. Its primary objective is to filter and optimize nodes and edges in a graph, aiming to mitigate the computational and storage overheads entailed by graph neural networks. This pursuit significantly bolsters the model's efficiency and scalability. Research has demonstrated that convolutional neural networks exhibit a significant abundance of redundant parameters spanning from the convolutional layer to the fully connected layer [38]. Consequently, the graph sparsification technique holds paramount significance in the realm of graph data processing. Presently, research on graph sparsification primarily concentrates on the following domains:

**Threshold Method:** This approach focuses on reducing the density of the graph by applying a threshold to filter the nodes or edges, classifying those below the threshold as invalid or insignificant. The careful choice of threshold is crucial to avoid information loss or model sparsity. Parameter adjustments should be tailored to the specific features of the problem and dataset when using thresholding methods. Jin et al. [14] proposed the Iterative Hard Thresholding method, which progressively reduces signal energy by applying a hard threshold to keep only the most significant components. This procedure gradually reduces the signal representation, preserving only the most energetic components. Zhou et al. [39] introduced the Forward-Backward Splitting method to address sparse constraints in loss functions, streamlining network training by avoiding complex operations such as second-order derivatives. However, predefining the sparsity may introduce bias in model selection, and both methods necessitate multiple iterations to attain the desired sparsity, leading to increased computational overhead.

**Figure Pruning Method:** The method trims the graph by sequentially removing edges with lower weights or insignificant nodes from the graph. Graph pruning can be achieved through various pruning techniques. LeCun et al. [40] proposed Optimal Brain Damage (OBD), which uses Hessian matrix second derivatives to prune less important weights. However, computing Hessian matrices is computationally intensive, and OBD may potentially over-prune weights, leading to a degradation of network performance. Hassibi et al. [41] proposed Optimal Brain Surgeon (OBS), which allows for unrestricted Hessian matrices and offers broader generalization capabilities than OBD. However, OBS can also become computationally expensive with large networks. Ding et al. [42] introduced ResRep method for lossless channel pruning in convolutional neural networks, maintaining performance while reducing layer width by re-parametrizing into a memorized and a forgotten component.

**Top-k method:** This method preserves the *k* edges with the highest weight or similarity in the graph while removing the remaining edges. The first *k* edges to be kept can be determined through weight ordering or utilizing other selection criteria. Lee et al. [43] proposed SAGPool, which employs a self-attention mechanism for top-k node selection to down-sample graphs, ensuring the preservation of crucial information while reducing size. Zheng et al. [16] proposed NeuralSparse for k-neighbor subgraph sampling, retaining only the top-k significant neighbors to mitigate computational overhead, and is applicable for node classification with GCN, GraphSage, or GAT. Despite its benefits, the top-k pruning technique may risk omitting vital information. Tai et al. [44] proposed the Spartan method, which also uses a top-k approach but incorporates a regularized optimal transmission problem for soft masking of low-volume parameters, facilitating adaptability to various sparsity allocation strategies.

**Sampling Method:** The method randomly selects certain nodes and edges while removing others through sampling. Sampling can be conducted using various techniques, including random sampling, uniform sampling, and node importance-based sampling. Rong et al. [45] proposed DropEdge as a method for achieving sparsification through random edge deletion in a graph. During each model training iteration, DropEdge stochastically removes input and output edges of each node based on a certain probability, thereby promoting the learning of a more resilient and generalizable representation by the model. However, the DropEdge method eliminates various edge subsets during distinct training iterations, while employing the entire graph for validation and testing. Nevertheless, the random removal of edges could potentially result in a loss of significant structural information that negatively impacts the model's performance. Zhang et al. [17] proposed the ANS-GT model, which adapts a multi-armed slot machine algorithm to sample attention nodes and select the most useful ones. However, it does not address the noise in the graph structure, which could affect downstream tasks. Fang et al. [18] proposed DropMessage as a method to mask GNN's feature of aggregation through message passing. The method performs a delete operation on the message matrix based on the delete rate, resulting in an element of the message matrix getting deleted during the propagation process.

In summary, despite the abundance of research on graph sparsification, none has been able to achieve a more precise and efficient sparsification method that reduces computational costs while accounting for the limitations of static attention.

### 2.3 Graph attention network

Velickovic et al. [24] integrated the attention mechanism into graph convolutional networks and introduced the GAT model, which demonstrated promising results in graph learning tasks, including node classification and link prediction. GAT utilizes an attention mechanism to learn importance weights for individual nodes, considering their graph position, attributes, and other properties, and subsequently aggregates information accordingly. Specifically, GAT leverages the attention mechanism at each node to ascertain its relative significance compared to other nodes, and subsequently calculates the weighted average of features from those nodes to derive a representation of the given node. However, recent studies indicate that GAT's attention computation (static attention) has limitations that hinder its ability to fit the training data [25]. In this research, we achieve dynamic attention by rearranging GAT's operations while retaining the same computational cost as GAT. Given the key vector K$=\left\{k_{1},k_{2},\ldots ,k_{m}\right\}\subset R^{d}$ and the query vector $Q=\left\{q_{1},q_{2},\ldots ,q_{n}\right\}\subset R^{d}$ in Dynamic Attention, the model posits a set of scoring functions $F=\left\{f_{1},f_{2},\ldots ,f_{k}\right\}$, where each scoring function f∈F is a mapping from $R^{d}\times R^{d}$ to R. If there is a mapping *φ*:[n]⟶[m] where a scoring function f∈F exists such that for any query vector $q_{i}\in Q$ and any key vector $k_{j}\in$ K where $j_{\neq \varphi \left(i\right)}\in \left[m\right]$, both satisfy $f\left(q_{i},k_{\varphi \left(i\right)}\right)\geq f\left(q_{i},k_{j}\right)$. In contrast, for every scoring function $f^{\prime }\in F^{\prime }$ in traditional static attention, there is a key $x_{f^{\prime }}\in \left[n\right]$ This key satisfies $f^{\prime }\left(q_{y},k_{xf^{\prime }}\right)\geq f^{\prime }\left(q_{y},k_{x}\right)$, where $q_{y}$ is any query vector in Q and $k_{x}$ is any key vector in K. In other words, the ranking of attention coefficients in static attention is not restricted by the query node, and the resulting ranking of attention weights remains consistent regardless of changes to the query node. To remove these constraints, this research employs a dynamic attention network within the model.

## 3 Model

**Figure 1:**
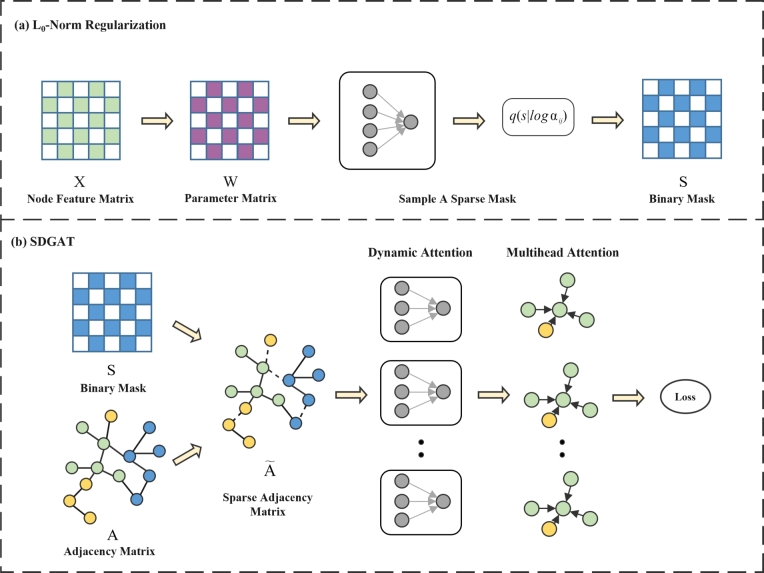
The SDGAT model: Panel (a) utilizes *L*_0_-norm regularization to generate binary mask *S*, where the input consists of node feature matrix *X*. Panel (b) combines the obtained binary mask *S* with the adjacency matrix *A* to construct a sparse adjacency matrix $\overset{˜}{A}$, and incorporates both a dynamic attention mechanism and multi-head attention mechanism.

This section provides a comprehensive description of the Sparse Graph-based Dynamic Attention Network (SDGAT). As Fig. 1(a) shown, the model first uses the $L_{0}$ norm regularization module to calculate the binary mask *S*, subsequently applying this mask to the original graph's adjacency matrix *A* as shown in Fig. 1(b) to produce a novel sparse adjacency matrix $\overset{˜}{A}$, thereby accomplishing the objective of eliminating noise from the graph's structure. So far, SDGAT has obtained a more concise graph structure through this sparsity. Subsequently, the model uses a dynamic attention network module to capture the interactions between different nodes more accurately. Furthermore, the model introduces a multi-head attention mechanism to capture diverse, vital information between nodes. This sequence of operations significantly enhances the model's generalization capability. The detailed notation related to the model is outlined below, forming the basis for the following discussions. Given a graph convolutional network G=(V,E,X), where *V* represents the set of *N* nodes, *E* denotes the set of edges, and $X=\left\{x_{1},x_{2},\ldots ,x_{N},\right\}$ denotes the matrix of node features. Set $h=\left\{\overset{\to }{h_{1}},\overset{\to }{h_{2}},\overset{\to }{h_{3}},\ldots ,\overset{\to }{h_{N}}\right\}$ to denote a set of features of the input *N* nodes, where $\overset{\to }{h_{i}}\in R^{F}$, with *F* as the feature dimension. This layer generates a fresh array of node characteristics (which may have distinct fundamentals $F^{\prime }$), $h=\left\{\overset{\to }{h_{1}^{\prime }},\overset{\to }{h_{2}^{\prime }},\overset{\to }{h_{3}^{\prime }},\ldots ,\overset{\to }{h_{N}^{\prime }}\right\}$ with each $\overset{\to }{h_{i}^{\prime }}\in R^{F^{\prime }}$ as its outcome. Denote the connectivity relationship as *A*, where $A_{ij}\in \left\{0,1\right\}$ indicates the presence or absence of an edge from node *i* to node *j*.

### 3.1 Graph structure sparsification

The objective of this module is to achieve a more logical graph structure through regularization of the input node features *X* with the $L_{0}$ approach, resulting in the computation of a binary mask *S*. Subsequently, using the computed binary mask, the model can make the initial graph structure sparser to create a new sparse graph. This step improves the rationality and accuracy of the diagram's structure.

In order to identify significant edges and exclude noise to form a new sparse graph, the model utilizes $s_{ij}\in \left\{0,1\right\}$ attached to each edge $e_{ij}\in E$. The use of $s_{ij}$ controls whether or not $e_{ij}$ will be used for neighbor aggregation. Moreover, the central node *i* is significant to itself, hence, the model assigns $s_{ii}$ as 1 to preserve its self-information. In this manner, the model can create a sparse graph that primarily comprises of only those edges that hold significance for the task at hand, thereby augmenting the precision and effectiveness of the model. More specifically, the model multiplies these binary masks by the adjacency matrix *A* to obtain a new edge-sparse adjacency matrix $\overset{˜}{A}$. The specific calculation process is illustrated in Eqs. (1), (2), and (3).

(1) $$\overset{˜}{A}=A⨀S$$

(2) $$S\in {\left\{0,1\right\}}^{m}$$

(3) $${\overset{˜}{A}}_{ij}=A_{ij}s_{ij\;}$$

where *m* represents the number of edges in the graph *G*, the model trains a binary mask *S* and training model parameters *W* through the minimization of the $L_{0}$ paradigm regularized empirical risk, as shown in Eqs. (4) and (5).

(4) $$R\left(S,W\right)=\frac{1}{n}\left(\sum_{i=1}^{n}L\left(y_{i},f\left(X,A⨀S,W\right)\right)\right)+\varphi ||S|{|}_{0}$$

(5) $$||S|{|}_{0}=\sum_{\left(i,j\right)\epsilon E}1_{\left[s_{ij}\neq 0\right]}$$

Here, *φ* is the regularized weight factor, L(⋅) corresponds to the loss function, f(X,A⨀S,W) is the attention-based aggregation function, and $||S|{|}_{0}$ denotes the number of $L_{0}$ paradigms (the number of nonzero elements in *S*) of the binary mask *S*. $1_{\left[s\right]}$ is an indicator function that equals 1 when condition *s* is met and 0 when it is not. The initial term refers to the empirical risk, while the latter denotes the regularization term. The optimization function enforces regularization constraints, driving redundant weights to zero through backpropagation. This creates a sparser trained network and leads to enhanced generalization performance.

### 3.2 Optimization formula

The computation of the gradient for Eq. (4), specifically the binary mask *S*, is essential for the model. However, since *S* comprises binary variables, neither the initial term nor the subsequent term can be considered on a microscopic scale. Hence, the model utilizes random variable optimization methods to effectively optimize the equations [26]. In this research, we consider each element $s_{ij}$ as a sample from the Bernoulli distribution Ber$\left(s_{ij}|\pi_{ij}\right)$, where $\pi_{ij}$ is a parameter of the Bernoulli distribution and can take any value between 0 and 1.

(6) $$\overset{ˆ}{R}\left(W,\pi \right)=E_{q\left(s|\pi \right)}\left[\frac{1}{n}\left(\sum_{i=1}^{n}L\left(y_{i},f\left(X,A⨀S,W\right)\right)\right)\right]+\varphi \sum_{(i,j)\epsilon E}\pi_{ij}$$

While the differentiability of the second parameter *π* in Eq. (6) has been achieved, the first parameter still presents a challenge. This difficulty arises from the computation of the expectation for a considerable number of binary random variables *S*, which makes efficient gradient computation problematic.

To solve this problem, the equation's gradient is estimated with a gradient estimator. Among various options, the hard concrete gradient estimator [46] is selected due to its high accuracy and efficiency in assigning hardened sparse binary masks. Compared to other gradient estimators, such as REBAR [47] and RELAX [48], the Hard Concrete Gradient Estimator used by the model converts the discrete problem into a continuous optimization problem. It does so by introducing a differentiable sigmoid function and a continuous noise distribution that map a sparse binary mask to a real number between 0 and 1. It also employs a Hard Concrete Function to restrict the mask range and prevent excessively sparse or overly dense issues. Specifically, a reparameterization technique approximates the optimization of the original problem through a similar proxy function. The calculations for the relevant parameters in Eq. (7) are shown in Eqs. (8), (9), and (10).

(7) $$\overset{˜}{R}\left(W,\log\alpha \right)=E_{u∼U(0,1)}\left[\frac{1}{n}\left(\sum_{i=1}^{n}L\left(y_{i},f\left(X,A⨀g(\overset{¯}{h}),W\right)\right)\right)\right]+\varphi \sum_{(i,j)\epsilon E}Sigmoid(\log\alpha_{ij}-\beta \log\frac{-\gamma }{\zeta })$$

(8) $$h=\;Sigmoid\left(\frac{\log u-\log\left(1-u\right)+\log\alpha }{\beta }\right)$$

(9) $$\overset{¯}{h}=h\left(\zeta -\gamma \right)+\gamma$$

(10) $$g\left(\overset{¯}{h}\right)=\min\left(1,\max\left(0,\overset{¯}{h}\right)\right)$$

Here, γ<0, ζ>1 and *β* are typical parameter values for hard concrete distributions. The concrete binary distribution is an expansion of the Bernoulli distribution and can approximate the Bernoulli distribution while optimizing its parameters through gradient-based reparameterization techniques. The value of *β* determines the approximation degree. If β=0, the original Bernoulli distribution is restored, but its differentiability is lost. If 0<β<1, a probability density is obtained, and the mass is concentrated near the endpoints. Thus, the distribution of hard concrete shares the same theoretical properties as the Bernoulli distribution. It can provide a more accurate estimation of the discrete properties by incorporating 0,1 into its support, while still permitting the optimization of its parameters through gradient procedures due to the continuous probability mass linking these two values. This distribution can be viewed as a “rounded” version of the original binary, where values greater than $\frac{1-\gamma }{\zeta -\gamma }$ are rounded to 1, and values less than $\frac{\gamma }{\zeta -\gamma }$ are rounded to 0.

**Figure 2:**
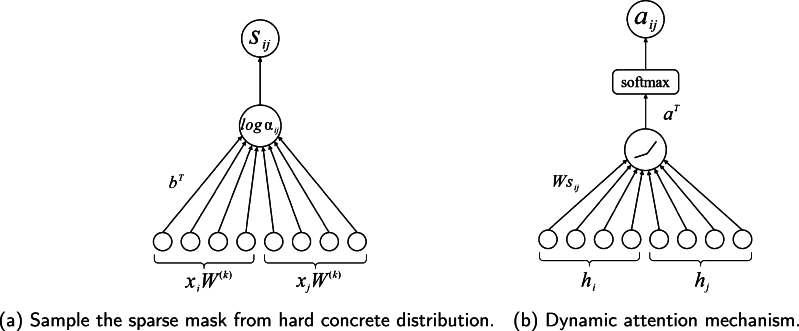
Schematic diagram of the model method.

During training, the model optimizes $\log\alpha_{ij}$ for each edge $e_{ij}$ in the graph. However, a more general approach is needed because this approach cannot generate new masks for edges that are not in the training graph. In this research, we utilized an inductive learning approach, specifically a generator model which utilizes the feature vectors of a node pair as input and generates a binary mask as output. To achieve this objective, the model will generate a generator for the log⁡α parameters in order to deduce inferences about new edges. This generator can be further trained to adapt to new data to produce more accurate masks, as shown in Fig. 2 (a). This allows the model to handle graph-structured data in a more general way while providing better predictions. One can simply model this generator as Eq. (11).

(11) $$\log\alpha_{ij}=(x_{i}W^{\left(k\right)}||x_{j}W^{\left(k\right)})b^{T}$$

where $W^{\left(k\right)}$ is the weight matrix of the *k*-th head and $b\in R^{D}$. The model defines the output of the generator as $\log\alpha_{ij}$ to integrate the generator into the end-to-end training pipeline. The model can sample the mask ${\overset{˜}{s}}_{ij}$ by feeding $\log\alpha_{ij}$ into the hard concrete distribution $q(s|\log\alpha_{ij})$. By using the set of mask sets $\overset{˜}{S}$ once more, it is possible to create a sparse edge graph that can be used for subsequent classification tasks.

During the testing phase, the model utilizes an estimator from Eq. (12) to generate deterministic masks.

(12) $$\overset{˜}{S}=\min\left(1,\max\left(0,\mathrm{Sigmoid}\;\left(\frac{\log(\alpha )}{\beta }\right)(\zeta -\gamma )+\gamma \right)\right)$$

This is the expected value of *S* given the hard concrete distribution q(S|log⁡α). ${\overset{˜}{s}}_{ij}$ is a continuous value between 0 and 1. Ideally, most elements of $\overset{˜}{S}$ will be zero, allowing for the removal of numerous edges from the graph and leading to a sparse graph.

### 3.3 Constructing dynamic attention networks

Traditional GAT applies a LeakyReLU nonlinearity to each node through a shared linear transformation, parameterized by the weight matrix *W*. This is illustrated in Eq. (13).

(13) $$e_{ij}=LeakyReLU(a^{T}•\left[Wh_{i}||Wh_{j}\right])$$

The primary issue with traditional GAT is the sequential application of the weight matrix *W* and the attention mechanism *a*, which may lead to their amalgamation into a single linear layer. To counteract this, SDGAT utilizes dynamic attention network [25], illustrated in Fig. 2 (b). The model first concatenates $h_{i}$ and $h_{j}$, then multiplies the concatenated vector with *W* and $s_{ij}$. After the product passes through a nonlinear activation layer, it is applied with *a* to obtain $e_{ij}$, as depicted in Eq. (14).

(14) $$e_{ij}=a^{T}LeakyReLU(W•s_{ij}•\left[h_{i}||h_{j}\right])$$

The model applies softmax function to normalize the attention scores, as depicted in Eq. (15).

(15) $$a_{ij}={\mathrm{softmax}}_{j}\;\left(e_{ij}\right)=\frac{\exp\left(e_{ij}\right)}{\sum_{k\in N_{i}}\exp\left(e_{ik}\right)}=\frac{\exp\left(a^{T}\mathrm{LeakyReLU}\;\left(W\cdot (h_{i}||h_{j})\right)\cdot s_{ij}\right)}{\sum_{k\in N_{i}}\exp\left(a^{T}\mathrm{LeakyReLU}\;\left(W\cdot (h_{i}||h_{k})\right)\cdot s_{ik}\right)}$$

The normalized attention coefficients are utilized to weigh the transformation characteristics of adjacent nodes. The weighted average serves as the new representation of node *i* by a nonlinear function *σ*, according to Eq. (16).

(16) $${\overset{\to }{h}}_{i}^{\prime }=\sigma \left(\sum_{j\in N_{i}}a_{ij}{\overset{\to }{h}}_{j}W\right)$$

Multiple attention mechanisms are also used in SDGAT to increase the capacity of the model. *K* independent attention mechanisms perform the transformations of the above equations and the model is computed using averaging to obtain the following output feature representation, as depicted in Eq. (17).

(17) $${\overset{\to }{h}}_{i}^{\prime }=\sigma \left(\frac{1}{K}\sum_{k=1}^{K}\sum_{j\in N_{i}}a_{ij}{\overset{\to }{h}}_{j}W_{k}\right)$$

where *K* represents the number of heads, $a_{ij}$ denotes the attention coefficient calculated by Eq. (15), and $W_{k}$ stands for the weight matrix of the *k*-th head.

## 4 Experiments

The research conducted a series of experiments to provide a comparative evaluation of SDGAT. This section initially elucidates the data employed in the experiments, the baseline model, and the pertinent parameter configurations. Subsequently, node classification experiments are executed to contrast the efficacy of the SDGAT model against other baseline node classification models. Furthermore, ablation experiments are conducted to substantiate the efficacy of the model's modules. To test the effect of different parameters on the model, thus the experiment changes some of the parameters. The experiment concludes with a visual representation of the nodes from several embedded methods and the model of this research to visually analyze the effectiveness of the different models on node classification.

### 4.1 Datasets

This experiment evaluates the SDGAT model using the real-world datasets: Cora, Citeseer and Pubmed [49], which are benchmark citation network datasets. Within these networks, each node corresponds to a paper, with edges indicating citation relationships between them. The node characteristics are important information about the paper and the labels are academic fields. The number of nodes, edges, feature dimensions, classes, training set size, validation set size, and test set size for these datasets are all summarized in Table 1.

**Table 1:** Statistics of the graph dataset used in the experiment, including the number of nodes, edges, feature dimensions, classes, training set size, validation set size, and test set size.

Dataset	#Nodes	#Edges	#Features	#Classes	#Training	#Validation	#Test
Cora	2708	13264	1433	7	140	300	1000
Citeseer	3327	12431	3703	6	120	500	1000
Pubmed	19717	108365	500	3	60	500	1000

### 4.2 Baselines

This experiment compares a number of state-of-the-art methods, including various classical graph neural network models. The baseline model is shown below:

• **GCN**[35]: This model is a scalable method for semi-supervised learning on graph-structured data, based on a variant of convolutional neural networks and allowing convolutional operations on graph-structured data. It aggregates neighbors by a local first-order approximation of the spectrogram convolution.
• **GAT**[24]: This model obtains node embeddings by efficiently aggregating neighbors through an attention mechanism. Each node in the graph structure can assign different weights to neighboring nodes based on their characteristics.
• **GraphSAGE**[50]: The core of the model's algorithm is to optimize the sampling of the whole graph to the sampling of the current neighboring nodes. Unlike other node representation learning methods, GraphSAGE is able to capture the contextual information of the nodes without relying on the global graph structure.
• **DropEdge**[45]: The essence of this model is the random removal of a certain number of edges from the input graph at each training epoch, which acts as a data enhancer and also as a message passing reducer. This model is compared in the experiments based on GAT.
• **SGAT**[26]: This model also uses sparse graphs based on GAT and simplifies its architecture by sharing a set of attention mechanisms across all layers of the model.
• **DropMessage**[18]: This model performs a drop operation on the propagated messages directly during message passing, stabilizes the training process by reducing sample variance, and preserves message diversity from an information-theoretic point of view. This model is compared in the experiments based on GCN and GAT.

The benchmark models were evaluated using their default configurations, as they typically yield optimal results. Experiments were performed using ReLU [51] as the activation function and the model was optimized using the Adam optimizer [52]. The model is a two-layer SDGAT, and the training times of the model was set to 200 times in the experiment, the learning rate is $l_{r}=1e-2$, the loss parameter of the $L_{0}$ regularization is set to φ=1e−6, and the number of multiple attention areas is K∈{2,3}. To ensure the fairness of the experiment, 10 repetitions of the experiment were performed on each of the different models and the final average was calculated.

### 4.3 Node classification

The experiment was conducted using various datasets to compare the performance of different models in node classification tasks. The results are presented in Table 2. The evaluation metrics ACC and F1 score are utilized to assess the model's performance. The last row of Table 2 shows the percentage increase or decrease in performance of SDGAT compared to the baseline model, which achieved the best performance. By analyzing the experimental results and comparing SDGAT with GAT, it can be observed that SDGAT significantly outperforms GAT on all datasets, especially on the Cora dataset where SDGAT outperforms GAT by about 3% on both evaluation metrics. In addition, SDGAT also outperforms DropMessage on both evaluation metrics on the Cora dataset. This indicates that utilizing the $L_{0}$ paradigm regularization technique and dynamic attention in the model for eliminating noise from the graph structure and acquiring information between nodes more efficiently than discarding passed messages operation directly, resulted in better performance. On the Citeseer dataset, SDGAT also achieved the best performance among all baselines, reaching 71.32% and 71.60% in the evaluation metrics of ACC and F1 score, respectively. In comparison to other popular embedding methods, the SDGAT model consistently demonstrates excellent performance on the Cora and Citeseer datasets. This suggests that the model's designed framework effectively enhances node classification performance in a robust manner. On the Pubmed dataset, SDGAT is notably superior to all GAT-based models. Although GCN-based models perform better on this dataset, the difference between SDGAT and GCN-based models in the two evaluation metrics is not significant. Overall, SDGAT holds a competitive advantage in performance.

**Table 2:** The node classification results of all models on three datasets, where the evaluation metrics are ACC and F1 score. Optimal results highlighted in bold.

Datasets	Cora		Citeseer		Pubmed	
Metrics	ACC	F1	ACC	F1	ACC	F1
GCN	79.60%	79.48%	70.58%	71.50%	79.78%	79.75%
GAT	82.34%	82.05%	70.35%	70.99%	77.53%	77.51%
GraphSage	83.04%	83.63%	70.06%	70.66%	78.30%	78.25%
DropEdge-GAT	81.96%	82.07%	70.62%	71.04%	77.55%	77.55%
SGAT	82.34%	82.09%	71.01%	71.45%	77.75%	77.70%
DropMessage-GCN	81.96%	82.30%	70.90%	71.34%	**79.91%**	**79.88%**
DropMessage-GAT	82.09%	82.13%	69.98%	70.27%	77.71%	77.67%
SDGAT	**85.29%**	**85.02%**	**71.32%**	**71.60%**	79.13%	78.86%

Intercomparison	+2.25%	+1.39%	+0.31%	+0.10%	-0.78%	-1.02%

### 4.4 Ablation experiment

**Figure 3:**
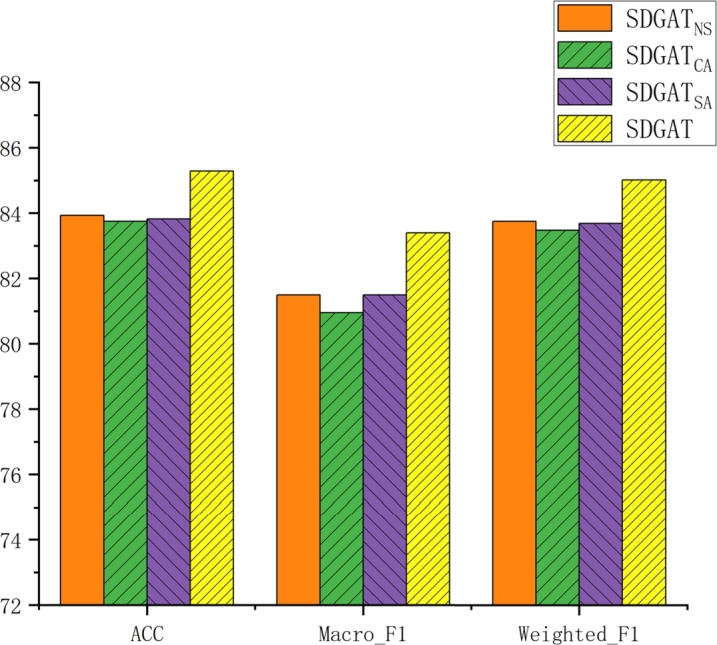
The results of the ablation experiments are presented, with the horizontal axis ranging from left to right indicating three evaluation metrics: ACC, Macro_F1, and Weighted_F1. The vertical axis represents the scores for these metrics.

To validate the effectiveness of each component of the SDGAT model in this research, as well as to verify whether SDGAT is similar to Ye and Ji [26], who postulated that the utilization of a set of attention coefficients is adequate for aggregating features. Three variants of SDGAT, SDGAT${}_{NS}$, SDGAT${}_{SA}$ and SDGAT${}_{CA}$ were designed and ablation experiments were performed on the Cora dataset to compare the performance of the different variants of the model, where SDGAT${}_{NS}$ removes the binary mask; SDGAT${}_{SA}$ replaces dynamic attention with static attention; and all layers in SDGAT${}_{CA}$ share the same attention mechanism. Fig. 3 summarizes the classification performance of SDGAT${}_{NS}$, SDGAT${}_{SA}$, SDGAT${}_{CA}$ and the original model SDGAT on the combined evaluation metrics Weighted_F1, Macro_F1 and ACC. The figure clearly demonstrates that SDGAT shows the most significant performance advantage over its three variants when evaluated using the Macro_F1 metric. In the remaining two metrics, the performance of these variants likewise does not surpass that of SDGAT, with the ACC for all three failing to exceed 84%. SDGAT${}_{NS}$ proves the validity of the sparse binary mask method and successfully removes the noise in the graph structure. By comparing the results with those of SDGAT${}_{SA}$, the experiment can conclude that SDGAT can make full use of the dynamic attention network, which has better results than the static attention network. In addition, the effect of SDGAT${}_{CA}$ further illustrates the effectiveness of the dynamic attention network in the SDGAT model, which can mine more rich and useful information from the multi-head attention at each layer.

### 4.5 Parameter sensitivity analysis

**Figure 4:**
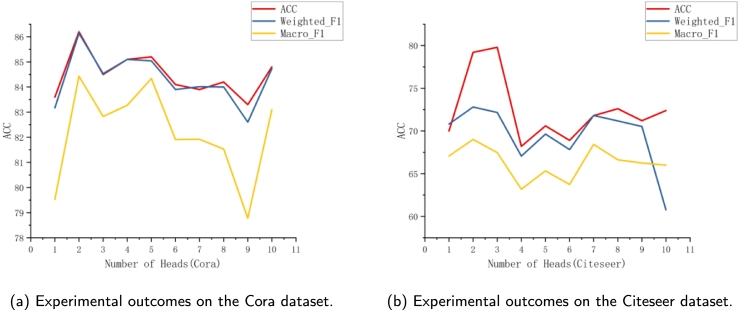
The impact of parameter *K* on the classification performance of the validation datasets for Cora (on the left) and Citeseer (on the right) is depicted. The horizontal axis represents the value of *K*, and the vertical axis indicates the scores for the three metrics: ACC, Macro_F1, and Weighted_F1.

In order to verify the effect of the parameter *K* in the model of this research on the model results, the parameter analysis was experimented on the datasets Cora, Citeseer to analyze the classification performance on the three composite evaluation metrics Weighted_F1, Macro_F1, and ACC. The parameter *K*, representing the number of attention heads, is pivotal in SDGAT, significantly influencing the model's learning capacity and susceptibility to overfitting. The appropriate value of *K* is selected in the experiments by the node classification accuracy observed on Cora and Citeseer datasets. The results are shown in Fig. 4(a-b), where the horizontal coordinate represents the number of attention heads *K*. With the parameter *K* ranging from 0 to 10, setting *K* to 1 disables the multi-head attention mechanism, leading to a degradation in model performance. At *K* values of 2 or 3, SDGAT attains the optimal or near-optimal performance across all evaluation metrics for the two datasets in question. However, as the number of attention heads *K* increases further, the model's performance declines again, and similar trends are observed on other datasets. Therefore, in the experiments of this research, the model chose to set *K* to 2 or 3.

**Figure 5:**
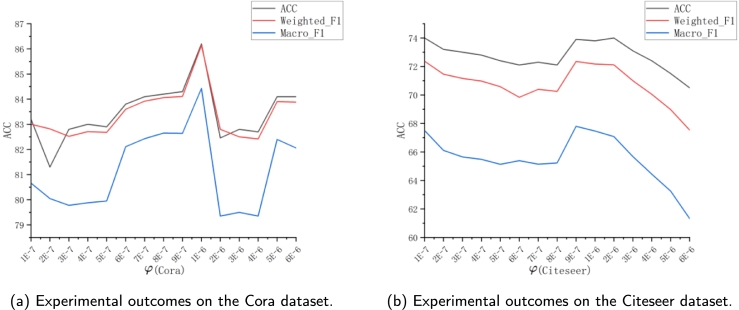
The impact of parameter *φ* on the classification performance of the validation datasets for Cora (on the left) and Citeseer (on the right) is depicted. The horizontal axis represents the value of *φ*, and the vertical axis indicates the scores for the three metrics: ACC, Macro_F1, and Weighted_F1.

Another important parameter is *φ*, which is used to balance the classification loss and edge sparsity. The experiments are also conducted on Cora and Citeseer to analyze the node classification performance on the three comprehensive evaluation metrics of Weighted_F1, Macro_F1 and ACC, and the specific results are shown in Fig. 5(a-b). By adjusting the size of *φ*, the model can control how much the model prunes the edges. Larger values of *φ* lead to stronger $L_{0}$ sparse regularization, thus pruning more edges. However, excessive pruning may remove some informative edges, leading to a decrease in classification accuracy. Therefore, the model selects an appropriate *φ* value to achieve the highest edge pruning rate while still maintaining good predictive performance for downstream classification tasks. As more edges are removed from the Cora dataset, the accuracy tends to increase when φ<1e−6, and the best results are achieved when φ=1e−6. There is a significant drop in accuracy when 1e−6<φ<4e−6, due to excessive pruning of the graph. For the same reason, the Citeseer dataset shows a drop in accuracy when φ>2e−6. It can be observed that both datasets achieve optimal or near-optimal performance when φ=1e−6, because the model chooses φ=1e−6 as the default value.

### 4.6 Visualization

**Figure 6:**
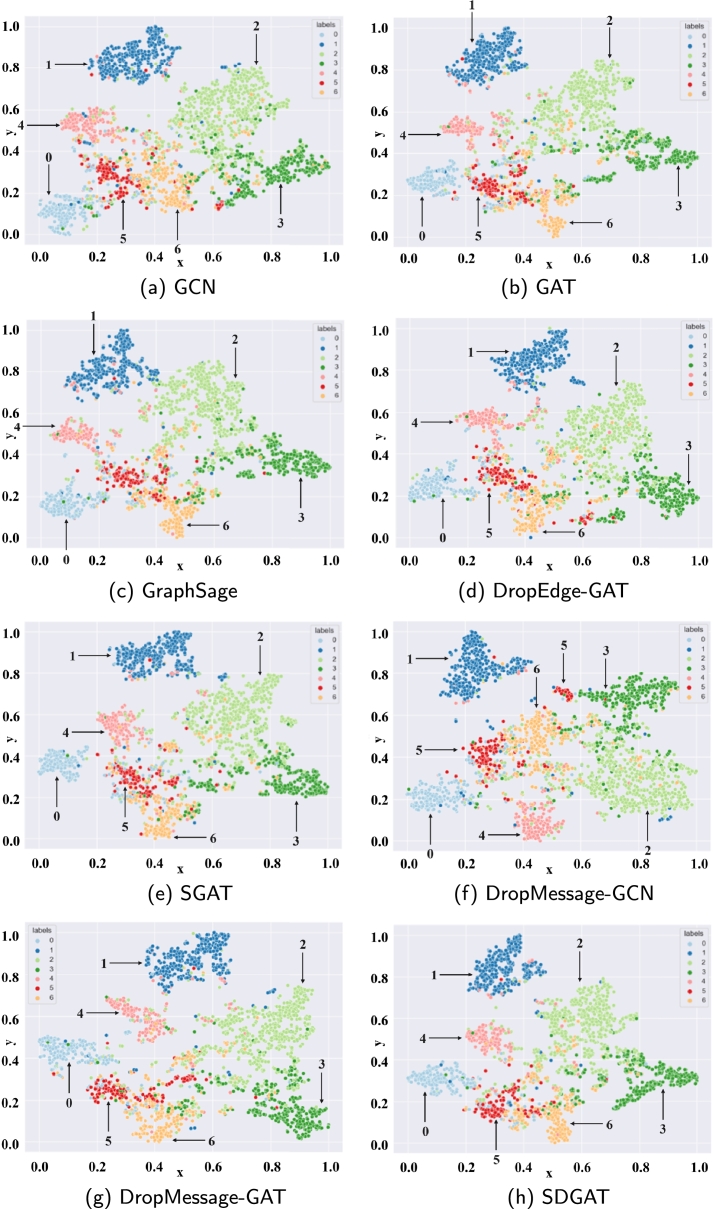
Cora dataset visualization. Panels (a) to (h) show different models, with unique numbers (colors) for node types.

To provide an intuitive understanding of the classification efficacy of the models examined in this research, alongside other node embedding models, this chapter utilizes a visualization method to depict the distribution of nodes within a lower-dimensional space. Fig. 6(a-h) displays the learned node embeddings of the different models in the Cora citation dataset for visual representation, and the learned embeddings are plotted using the PCA approach, where different samples are visualized with different colors. In comparison to GAT, SDGAT demonstrates larger inter-class distances and a superior ability to differentiate between node types. For instance, when examining the comparison between the second and fourth category in Fig. 6(h), SDGAT showcases more transparent boundaries. Compared to the other models, it is intuitively observed that SDGAT is better able to discriminate between different node types and has tighter distances within nodes. For example, compared to the first category of nodes in Fig. 6(g), the first category of nodes in SDGAT are more closely connected. This indicates that the combination of $L_{0}$ norm regularization and dynamic attention networks can robustly enhance the performance of node classification.

## 5 Discussion

Most popular GNN models are not effective at handling noise in real graph structures. This causes noise to propagate and ultimately impacts downstream tasks. Additionally, traditional static attention mechanisms limit the effective acquisition of information among nodes. Our research proposes SDGAT as a solution to these issues, providing effective problem-solving measures. Compared to traditional sparsification studies, SDGAT clearly outperforms them. However, there are deficiencies in current sparse research studies. For instance, DropMessage [18] randomly deletes delivered messages, which cannot precisely remove noise from the graph structure. Additionally, most existing graph sparsification techniques primarily rely on traditional static attention, which does not further explore the information within the graph. The dynamic attention module introduced in SDGAT can well compensate for this defect, making the interaction information between nodes in the sparsified graph structure fully utilized. Each layer of the attention mechanism can obtain more effective information and does not cause too much computational burden. However, the current SDGAT is only capable of removing noise within the graph structure and does not further address the absence of nodes and edges in the graph itself. Future research may consider integrating methods of graph structure learning to tackle this challenge. In the node classification experiments, our model achieved the best performance on both the Cora and Citeseer datasets, particularly in Cora, where it significantly outperformed other baselines. On the Pubmed dataset, while SDGAT significantly outperforms GAT-based models, GCN-based models still outshine it. Although the gap between SDGAT and GCN-based models in terms of ACC and F1 score is not pronounced, we plan to further optimize the model. For example, we can explore the introduction of GCN into the model and adaptively integrate the information obtained, aiming to maintain robust performance across a variety of datasets.

## 6 Conclusion

In this paper, we propose a method called SDGAT, aiming at mitigating the model's reliance on the original graph structure. SDGAT designs a dynamic attention module founded on graph sparsification. This empowers the model to effectively extract interactional information amidst nodes, while simultaneously eliminating noise and redundant data within the graph structure. This methodology enhances the model's capability to capture authentic signals and curtails overfitting to noise. Furthermore, the dynamic attention mechanism enables the model to adaptively fine-tune attention weights and selectively concentrate on paramount graph nodes and edges within the graph structure, so that each layer of attention to extract valid information. In this research, SDGAT was evaluated on three real citation networks for node classification experiments and compared with the results of other baselines. Additionally, through ablation experiments, parameter sensitivity analysis, and visualization, we further substantiated the exceptional performance of the SDGAT model.

## CRediT authorship contribution statement

**Runze Chen:** Writing – review & editing, Writing – original draft, Visualization, Methodology, Conceptualization. **Kaibiao Lin:** Writing – review & editing, Validation, Resources, Funding acquisition. **Binsheng Hong:** Data curation, Conceptualization. **Shandan Zhang:** Writing – review & editing, Writing – original draft, Investigation. **Fan Yang:** Writing – review & editing, Formal analysis.

## Declaration of Competing Interest

The authors declare that they have no known competing financial interests or personal relationships that could have appeared to influence the work reported in this paper.

## Data Availability

The data used in this research was obtained from the Deep Graph Library (DGL) public database.
